# A Kalman Filter-Based Method for Diagnosing the Structural Condition of Medium- and Small-Span Beam Bridges during Brief Traffic Interruptions

**DOI:** 10.3390/s20154130

**Published:** 2020-07-24

**Authors:** Qingfei Gao, Xiang Wang, Yang Liu

**Affiliations:** 1 School of Transportation Science and Engineering, Harbin Institute of Technology, Harbin 150090, China; gaoqingfei@hit.edu.cn; 2 China Railway Bridge Science Research Institute, Ltd., Wuhan 430034, China; lwangxiang4143@163.com; 3 State Key Laboratory for Health and Safety of Bridge Structures, Wuhan 430034, China

**Keywords:** condition diagnosis, beam bridges, Kalman filter, novelty detection

## Abstract

Load tests are a popular way to diagnose the structural condition of bridges, however, such tests usually interrupt traffic for many hours. To address this issue, a Kalman filter-based method is proposed to diagnose the structural condition of medium- and small-span beam bridges by using the acceleration responses obtained from the bridge during a brief traffic interruption. First, a condition diagnosis feature based on the Kalman filter innovation (i.e., the optimal difference between the filter predictions and measured responses) is presented. Second, a condition diagnosis index, which is the energy ratio between the innovation and the measured acceleration, is generated by calculating the null space of the Hankel matrix consisting of condition diagnosis features. Then, on the basis of the novel detection, a method is used to diagnose the structural condition of a bridge during a brief traffic interruption. Finally, the validity and dependability of the proposed method is demonstrated through experimental tests with a model bridge and field tests on an actual bridge. Using the proposed method, the long-time interruption of traffic flow and the reliance on finite element model are effective avoided during the process of condition diagnosis of bridges.

## 1. Introduction

The structural responses of bridges during operation can be used for condition diagnosis, wherein the main tasks are evaluating the safety status of bridges and identifying potential damage. Visual inspection [1,2,3,4,5], which manually checks the safety status of bridges by measuring cracks, defects and leakage, is an easy and popular way to assess the condition of bridges, however, the accuracy and quality of visual inspection diagnosis results are subjective and dependent on the diligence of the inspectors. Compared with visual inspection, structural health monitoring is a technique that continuously provides large amounts of reliable structural response data [6,7,8,9,10,11], thus, this technique is an effective way to assess the condition of bridges. However, it is economically infeasible to establish a structural health monitoring system for all bridges, especially for medium- and small-span bridges, which account for a large proportion of all bridges. To date, the load test is the most effective and the most widely used approach for condition diagnosis in medium- and small-span bridges [12,13].

To accurately diagnose the bridge technical condition, there are two categories of load tests, including the diagnostic load test and the proof load test [14,15,16,17,18]. For the former, a multi-grade load is implemented to a bridge until the predetermined safe load is attained. For the latter, a lower magnitude load is usually adopted, and the measured responses of bridge are used to assess the structural condition of bridges. For the load test, the loading vehicle is usually a standard truck combined with a uniform load, which is defined by the axle load and spacing. Usually, the issue in proof load testing is that the traffic flow must be interrupted for many hours to ensure the loading time is sufficient for each load grade.

In this study, a diagnostic load test with a brief interruption in traffic flow is presented. The daily traffic of a bridge is interrupted first. Then, the loading vehicle travels across this bridge at a constant speed, and the acceleration response of the bridge superstructure induced by the loading vehicle is acquired. The abovementioned load procedure can be repeated several times by ensuring that the loading vehicle travels along the same driving route at the same speed. Finally, after completing the entire load test, the traffic resumes again. The abovementioned procedure usually does not require much time for medium- and small-span bridges. The measured acceleration response is applied to diagnose the condition of bridges by using a Kalman filter-based method.

Kalman filters were originally widely applied in statistical analysis and time series analysis [19,20,21,22,23]. This filter is composed of a group of recursive equations that provide a simple way to update and predict the response in a state space model of structures. Generally, the Kalman filter establishes the optimal predictor which is a linear model with limited parameters, using the least squares method. Utilizing the characteristics of the Kalman filter, the optimal differences between the measured response and the filter predictions, named innovations, are used to detect damage in structures [24] by testing the hypothesis on the whiteness of the innovation. Different types of Kalman filters, including adaptive Kalman filters [25,26], extended Kalman filters [27,28,29], unscented Kalman filters [30,31] and dual extended Kalman filters [32,33], have been investigated to diagnose structural damage. Additionally, some methods have been combined with the Kalman filter to enhance the ability to detect structural damage. Based on energy theory, a relationship between the structural stiffness and acceleration response was generated by using Kalman filter, and this relationship was applied to fast detect the damage of structures [34]. An extended Kalman filter-based artificial neural network was proposed to detect the damage in bridges caused by the changes of environmental temperature [35]. As previously discussed, the Kalman filter is an effective method to directly utilize the acceleration response to diagnose the change in the condition of structures.

In this study, Kalman filter is combined with the diagnostic load test with a brief interruption in traffic flow. After performing the diagnostic loading tests, a Kalman filter-based method is adopted to diagnose the condition of the bridge using the measured acceleration responses of the bridge superstructure. With this way, a long duration interruption of the traffic flow during the bridge condition diagnosis process is effectively avoided. Using the Kalman filter, the measured acceleration responses of bridge superstructure are directly utilized to assess the structural condition of the bridge, so it effectively avoids the calculation errors of data secondary processing such as modal parameter identification using acceleration data. Additionally, the proposed Kalman filter-based method does not depend on the finite element model (FEM) of bridge; thus, this method also avoids the errors caused by any difference between the analytical FEM of the bridge and the real structural characteristics of the bridge.

## 2. A Kalman Filter-Based Method for Diagnosing the Structural Condition of Medium- and Small-Span Beam Bridges

In this section, a method is presented to diagnose the condition of a bridge during a brief interruption in traffic flow by using the acceleration response of the bridge superstructure induced by the action of a moving vehicle traveling across the bridge at constant speed. A condition diagnosis feature is generated by using the innovation between the measured acceleration and the predicted acceleration obtained from the Kalman filter, and then a condition diagnosis index—which is the energy ratio between the innovation and the measured acceleration—is proposed by calculating the null space of the Hankel matrix consisting of condition diagnosis features. Following the basic novel detection idea, the established condition diagnosis index is utilized to assess the condition of the bridge.

### 2.1. Condition Diagnosis Feature Based on the Innovation Obtained by the Kalman Filter

A bridge is deemed a linear structural system. Thus, on the basis of state space theory, the state equation of a linear discrete system of a bridge is defined as follows:

(1) $$x_{k+1}=Ax_{k}+w_{k}$$

(2) $$y_{k}=Cx_{k}+v_{k},$$

where *k* represents the sampling time point of the structural response (k=1,2,⋯,N), in which *N* is the count of sampling time points; $x_{k}\in R^{n\times 1}$ represents the state vector at the *k*th sampling time point, wherein *n* is the system order, which is twice the total number of degrees of freedom of bridge; $x_{k+1}\in R^{n\times 1}$ is the state vector at the (*k+*1)th sampling time point; $A\in R^{n\times n}$ is the system matrix; $y_{k}\in R^{m\times 1}$ is the output vector of the structure at the *k*th time point; *m* is the total number of measured degrees of freedom; $C\in R^{m\times n}$ is the state output matrix; $v_{k}\in R^{m\times 1}$ is the measured noise vector at the *k*th time point; and $w_{k}\in R^{n\times 1}$ is the noise of the excitation load. The noise of the excitation load is defined by the following equation:

(3) $$w_{k}=G\rho_{k},$$

where $\rho_{k}\in R^{r\times 1}$ is the unmeasured excitation vector at the *k*th time point, *r* is the total number of excitation loads, and $G\in R^{n\times r}$ is the transfer matrix between the input load and state vector of the system. The vector $v_{k}$ is defined by the following equation:

(4) $$v_{k}=D\rho_{k}+\eta_{k},$$

where $D\in R^{m\times r}$ is the transmission matrix and $\eta_{k}\in R^{m\times 1}$ is the pure measured noise at the *k*th time point.

The innovation is defined as the optimal differences between the measured and predicted responses of the bridge obtained by the Kalman filter [24]. Based on the abovementioned equations, the innovation $e_{k}$ is computed as follows:

(5) $$e_{k}=y_{k}-C{\hat{x}}_{k}^{-},$$

where ${\hat{x}}_{k}^{-}$ is the prior state estimation vector at the *k*th time point. The posterior state estimation vector at the *k*th time point can be estimated by using the Kalman filter [36], defined as follows:

(6) $${\hat{x}}^{+}{}_{k}={\hat{x}}_{k}^{-}+\bar{K}e_{k},$$

where $\bar{K}$ is the steady state Kalman gain, which is computed by using the following equation:

(7) $$\bar{K}=PC^{T}{\left(CPC^{T}+R\right)}^{-1},$$

where the covariance matrix of the state error $P\in R^{n\times n}$ is defined as follows:

(8) $$P=\bar{A}P{\bar{A}}^{T}+A\bar{K}R{\bar{K}}^{T}A^{T}+Q,$$

(9) $$\bar{A}=A(I-\bar{K}C),$$

As described above, the innovation obtained by the Kalman filter is related to the measured responses of bridges, and the measured responses are directly determined by the excitation load acting on the bridge. For a bridge without any damage, the Kalman filter is obtained by using the measured acceleration of the bridge superstructure under the action of a certain excitation load, and the innovation e is calculated by using the generated Kalman filter. If the same excitation load acts on this bridge, another innovation $e^{\prime }$ is acquired by using the abovementioned generated Kalman filter. If the structural condition of this bridge does not change, the two innovations, e and $e^{\prime }$, should be the same in theory. Therefore, the innovations obtained from the same excitation load can be used to diagnosis the condition of the bridge.

However, for a bridge in operation, it is impossible to keep the excitation load consistent for different times. To solve this issue, a reasonable method is to stop the traffic and to excite the bridge by using the same standard loading vehicle traveling along the same driving route at the same constant speed for every load test. This excitation method is similar to the regular load test of a bridge, and it is easy to implement for real situations. In contrast to the regular load test, we do not need to stop the traffic flow for many hours for each load test because for the selected medium- and small-span beam bridges, the loading vehicle does not require much time to travel across the bridge even though the vehicle speed is very low. As discussed above, the innovations obtained by the Kalman filter using the acceleration responses of the bridge are defined as the condition diagnosis feature. These innovations form the following matrix:

(10) $$e={\left[\begin{array}{cccccc} e_{1} & e_{2} & \cdots & e_{j} & \cdots & e_{m} \end{array}\right]}^{Τ}=\left[\begin{array}{cccc} e_{1,1} & e_{2,2} & \cdots & \begin{array}{ccc} e_{k,1} & \cdots & e_{N,1} \end{array}\\ e_{1,2} & e_{2,2} & \cdots & \begin{array}{ccc} e_{k,2} & \cdots & e_{N,2} \end{array}\\ \vdots & \vdots & \ddots & \vdots \\ e_{1,m} & e_{2,m} & \cdots & \begin{array}{ccc} e_{k,m} & \cdots & e_{N,m} \end{array} \end{array}\right],$$

where *j* (j=1,2,⋯,m) is the total number of measured acceleration responses of the bridge.

### 2.2. Condition Diagnosis Index Based on the Energy Ratio between the Innovation and the Measured Response

The energy ratio calculated by using the measured acceleration response and its corresponding innovation is defined by the following equation:

(11) $$\beta_{j}=\frac{\left(e_{j}\right){\left(e_{j}\right)}^{T}}{\left(y_{j}\right){\left(y_{j}\right)}^{T}},$$

where $\beta_{j}$ is the energy ratio between the innovation and the acceleration response at the *j*th measured point and $y_{j}=\left\{\begin{array}{cccccc} y_{1,j} & y_{2,j} & \cdots & y_{k,j} & \cdots & y_{N,j} \end{array}\right\}$ is the acceleration response at the *j*th measured point. Using Equation (11), the energy ratio of all the measured points can be obtained, and then the following vector is formed:

(12) $$\beta =\mathrm{sort}\left(\left\{\beta_{1},\beta_{2},\cdots ,\beta_{j},\cdots ,\beta_{m}\right\}\right),$$

where $\mathrm{sort}\left(\cdot \right)$ is the operator of arranging the order from small to large. The vector β contains all the information from the innovations of the predicted acceleration responses. The change in β is the key to diagnosing the variation in the structural condition of a bridge. To obtain consistent and comparable results every time, the vector β should be reordered. In this study, it is recommended to arrange the vector β so that the values change from small to large. After acquiring the vector β, the following Hankel matrix, denoted as ***H***, can be formed:

(13) $$H=\left[\begin{array}{cccc} \beta_{1} & \beta_{2} & \cdots & \beta_{q}\\ \beta_{2} & \beta_{3} & \cdots & \beta_{q+1}\\ \vdots & \vdots & \ddots & \vdots \\ \beta_{p} & \beta_{p+1} & \cdots & \beta_{p+q-1} \end{array}\right],$$

where p and q are the number of rows and columns in the Hankel matrix (p<q), respectively.

The elements on the antidiagonal of the Hankel matrix are the same, i.e., two adjacent columns are misaligned. Therefore, any column of the Hankel matrix has a significant correlation with each other, which can be selected as a delay vector as follows:

(14) $$B_{q}={\left\{\beta_{q},\beta_{q+1},\cdots ,\beta_{p+q-1}\right\}}_{}^{Τ}\in {\mathbb{R}}^{p\times 1},$$

According to Equation (13), the length of the delay vector is p, and the number of delay vectors is q. Because adjacent delay vectors are highly correlated, when p is very small, q increases, and the correlation between any two delay vectors decreases. In contrast, if p is large, q decreases, and the correlation between each delay vector increases.

In this study, the first load test of a bridge is defined as the reference condition, and the corresponding Hankel matrix under the reference state is named $H_{}^{0}$. The following equation is obtained:

(15) $$H_{}^{0}N_{}^{0}=0,$$

where $N_{}^{0}\in {\mathbb{R}}^{q\times 1}$ is any column vector of the right null space of matrix $H_{}^{0}$ in the reference state. This column vector is defined as follows:

(16) $$N_{}^{0}=\mathrm{column}\left(\mathrm{null}\left(H_{}^{0}\right)\right),$$

where $\mathrm{null}\left(\cdot \right)$ is the operator of calculating the right null space of the matrix and $\mathrm{column}\left(\cdot \right)$ is the operator of taking any column of one matrix.

In addition to the reference condition, the load test is repeated $m_{H}$ times under the condition of bridge without any structural damage, and we intuitively define these tests as the load tests under the healthy condition of bridge. For the bridge without any damage, the Hankel matrix obtained by using the $k_{H}$th test is denoted as $H_{k_{H}}$. Using the generated null space $N_{}^{0}$ under the reference condition, the following residual $\alpha_{k_{H}}$ ($\alpha_{k_{H}}\in {\mathbb{R}}^{p\times 1}$) is obtained from the following equation:

(17) $$\alpha_{k_{H}}=H_{k_{H}}N_{}^{0},$$

where $\alpha_{k_{H}}$ is the vector of residuals under the healthy condition of the bridge and $k_{H}\in \left(1,\;2,\;\cdots ,\;m_{H}\right)$ is the number of load tests.

Theoretically, for the reference condition and the healthy condition defined above, $H_{k_{H}}$ is the same as $H_{}^{0}$; thus, $\alpha_{k_{H}}$ is a perfect zero vector. However, owing to measurement noise, the residual values cannot be zero but are close to zero. Because the number of repeated load tests is small and no statistical characteristics are available, the condition diagnosis index is defined to evaluate the vector of residual. This index is expressed as follows:

(18) $$\gamma_{k_{H}}=\left\|\alpha_{k_{H}}\right\|=\sqrt{\alpha_{k_{H}}^{T}\cdot \alpha_{k_{H}}},$$

where $\gamma_{\;k_{H}}$ is the condition diagnosis index of the $k_{H}$th load test. After obtaining all the condition diagnosis indexes of $m_{H}$ loading tests under the healthy condition of the bridge, the following threshold under the healthy condition of the bridge is defined:

(19) $$\eta =\theta \cdot \frac{1}{m_{H}}\sum_{k_{H}=1}^{m_{H}}\gamma_{k_{H}},$$

where η is the threshold under the healthy condition of the bridge and θ is the guarantee coefficient. Usually, the value of guarantee coefficient should be determined case by case. This value depends on the test condition, the ratio of signal to noise of measured data, pavement situation etc. For the example of actual bridge described in Section 4, the guarantee coefficient is taken 1.2 according to the real condition of load test.

Except for the abovementioned two conditions, all the other conditions of bridge are defined as the condition to be diagnosed. The residual of the *z*th loading test is calculated by using the following equation:

(20) $$\alpha_{z}=H_{z}N_{}^{0},$$

where $\alpha_{z}$ is the vector of residuals for the condition to be diagnosed and *z* is the number of load tests. The condition diagnosis index for the condition to be diagnosed is calculated by using the following equation:

(21) $${\gamma^{\prime }}_{z}=\left\|\alpha_{z}\right\|=\sqrt{\alpha_{z}^{T}\cdot \alpha_{z}},$$

where ${\gamma^{\prime }}_{z}$ is the condition diagnosis index of the *z*th load test. If ${\gamma^{\prime }}_{z}$ is larger than η, the condition of the bridge is deemed abnormal. Conversely, the bridge is considered healthy if ${\gamma^{\prime }}_{z}$ is smaller than η.

For a bridge in actual operation, especially for newly constructed bridges, it is appropriate to regularly perform the proposed method to diagnose the condition of bridges. With the accumulation of load test data, the statistical characteristics of condition diagnosis indexes can be obtained. Thus, the threshold for the healthy condition of the bridge can be calculated by using a statistical approach. In this way, the robust performance of the proposed method is enhanced. For the proposed method, the results of condition diagnosis do not need to consider the influence of environmental temperature because the time required for a load test is about half an hour on average. However, the environmental temperature of load test for different time should be similar, so the results obtained by different load tests could be compared.

As discussed above, the proposed Kalman filter-based method does not need to establish the FEM of the bridge, so the calculation errors caused by the differences between the analytical FEM and the structural performance of the actual bridge are avoided during the bridge condition diagnosis process. Additionally, compared with other types of structural responses, it is easy to measure the acceleration response and to ensure the high accuracy of the data. Therefore, it is relatively convenient for the practical application of proposed method.

### 2.3. Procedure of the Proposed Method

**Figure 1:**
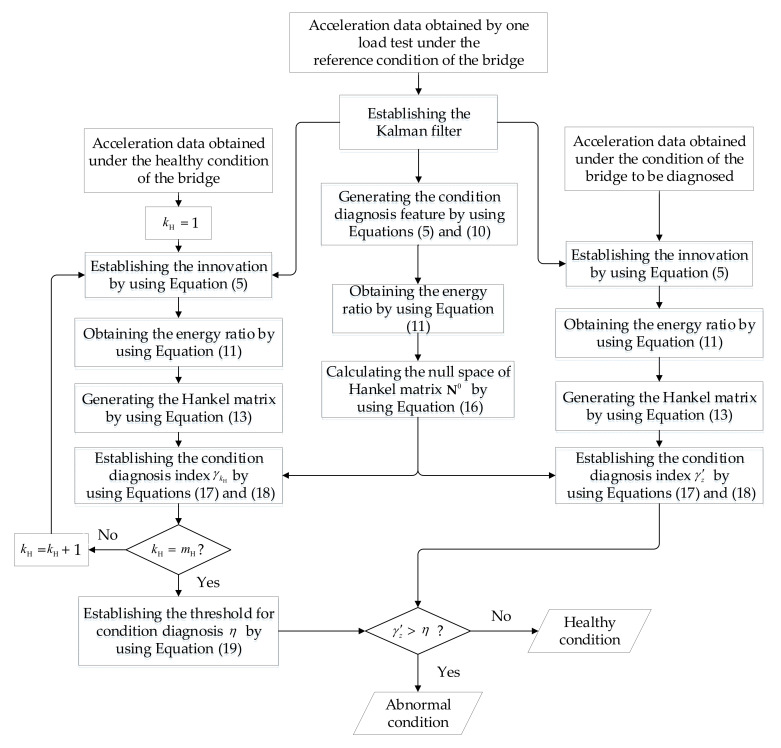
Flowchart of the proposed Kalman filter-based method for diagnosing the condition of bridges.

Under the reference condition, the Kalman filter, the Hankel matrix $H_{}^{0}$ and its null space $N_{}^{0}$ are established by using the acceleration responses of bridge. With the measured data under the healthy condition, the condition diagnosis index $\gamma_{\;k_{H}}$ and the threshold η are calculated. For the condition to be diagnosed, a condition diagnosis index ${\gamma^{\prime }}_{z}$ is obtained by using $N_{}^{0}$ and the measured data. If the value of ${\gamma^{\prime }}_{z}$ surpasses the threshold η, the abnormal condition of the bridge will be determined; on the contrary, the condition of the bridge is believed to be safe. The whole procedure is shown in Figure 1.

## 3. Example of an Experimental Model Bridge

### 3.1. Description of the Model Bridge Experiment

**Figure 2:**
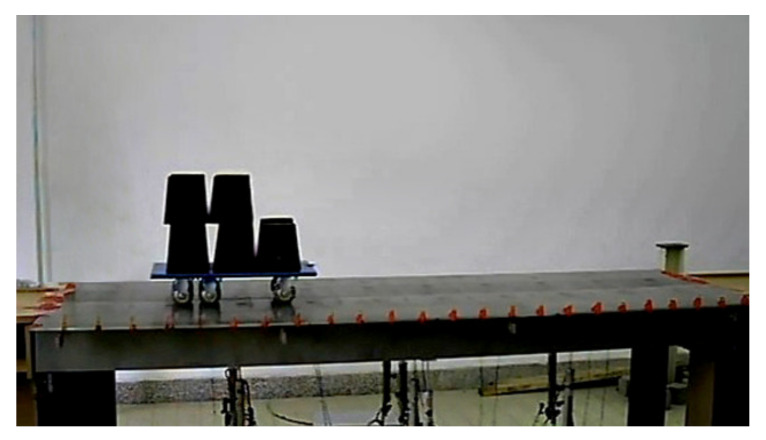
Photograph of the whole experimental system.

The model is a simply supported beam whose cross-section is composed of several T-shaped steel beams, and the detailed information of the loading vehicle and the material parameters and geometrical size of the bridge are given in the literature [37]. A photo of the whole experimental system is shown in Figure 2. A total of 10 accelerometers (PCB Group, Inc., Depew, NY, USA) are installed on two T-shaped beams to measure the response induced by the moving load. The frequency range of each accelerometer works from 0 to 80 Hz, and the measurement range is ±2 g pk. The sensitivity of each accelerometer is 100 mv/(m/s^2^), and all the technical specifications of this type of sensor satisfy the test needs.

**Figure 3:**
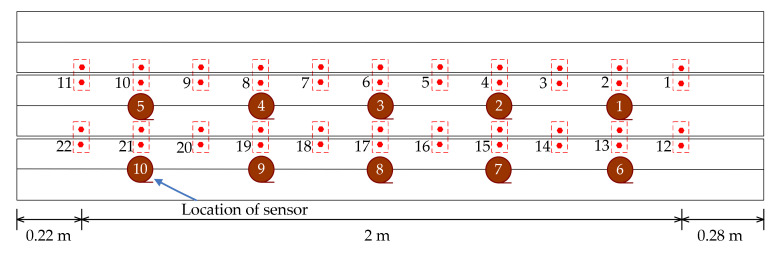
Arrangement of the accelerometers and the devices simulating damage in the transverse connections.

**Figure 4:**
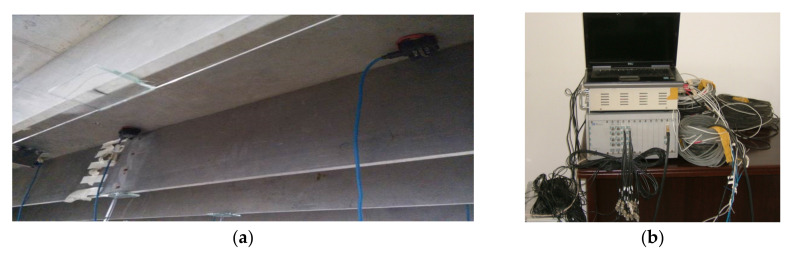
Photographs of the sensing system: (**a**) accelerometers; (**b**) data acquisition device.

A schematic of the sensor placement is shown in Figure 3. Another 22 devices are designed to simulate the damage of the transverse connections between the two T-shaped beams, as shown in Figure 3. A SCADAS III data acquisition system (LMS Company, Leuven, Belgium) is used to acquire the acceleration signals. The photos of accelerometers and data acquisition system are shown in Figure 4.

A total of 22 experimental cases were implemented to verify the validity and dependability of the proposed method. The healthy model bridge was excited by a 120 kg moving load traveling at a constant speed, and this process was repeated a total of nine times; the corresponding cases are denoted Case 1 through Case 9, as shown in Table 1. Another two different damage conditions for this model bridge were implemented, and the corresponding cases are denoted Case 10 through Case 15. Finally, two types of structural conditions for this model bridge were generated by changing the weight of the moving load, as shown in Table 1.

### 3.2. Results of Condition Diagnosis of the Model Bridge Obtained by the Proposed Method

**Figure 5:**
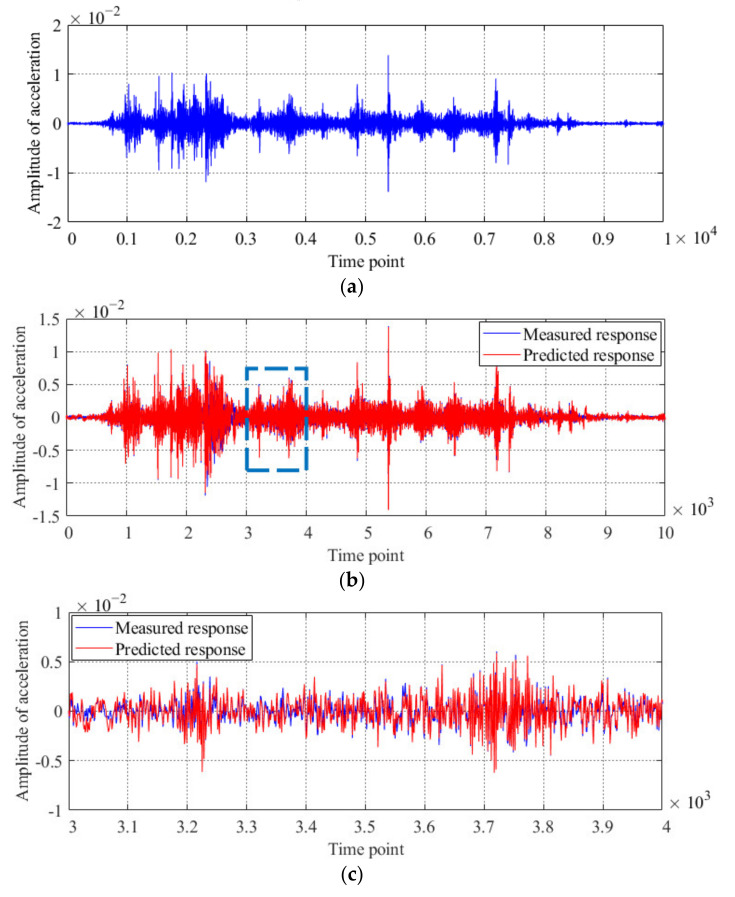

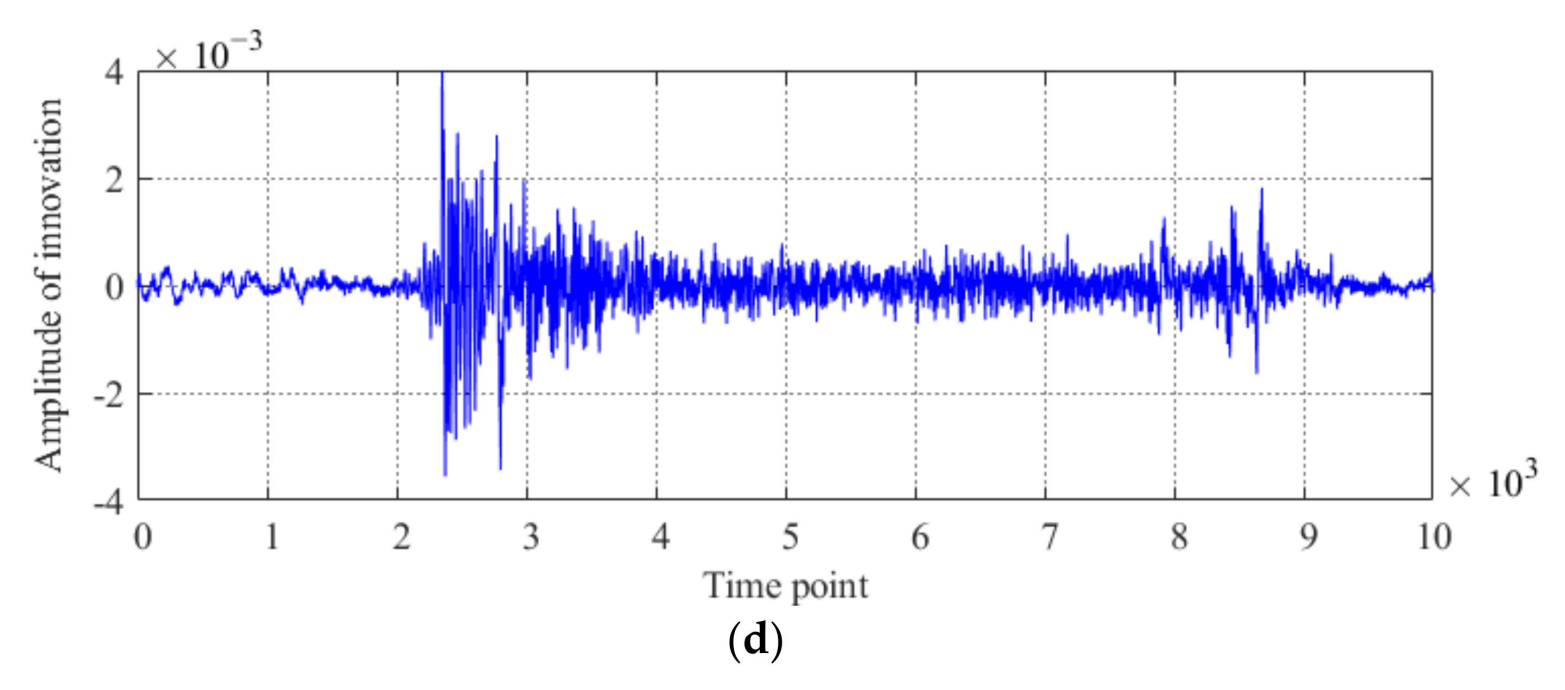
Results of the Kalman filter obtained by using the acceleration response of the model bridge acquired from Case 1: (**a**) measured acceleration response of sensor #3; (**b**) comparison between the measured and predicted acceleration responses of sensor #3; (**c**) detailed parts of the innovation from time points 3000 to 4000, and (**d**) innovation obtained by using the acceleration response of sensor #3.

During the test process, as the moving vehicle travels across the bridge at a constant speed, the acceleration responses of the superstructure of this model bridge are acquired with the SCADAS III data acquisition system. The sampling frequency for each test is set to 400 Hz, and the time history of the acceleration response obtained by sensor #3 (Case 1) is shown in Figure 5a. All the acceleration responses obtained from Case 1 are implemented to generate the Kalman filter of the healthy condition of this model bridge. With the generated Kalman filter, the predicted acceleration is obtained. A comparison between the predicted and measured accelerations is shown in Figure 5b–c. As described in Equation (5), the innovation, a unique concept in Kalman filter, which can be obtained as the optimal difference between the filter predictions and the measured acceleration response of sensor #3, is shown in Figure 5d. From the results in Figure 5, it is deduced that the prediction accuracy of the generated Kalman filter is sufficient to satisfy the requirements for diagnosing the condition of the model bridge.

**Figure 6:**
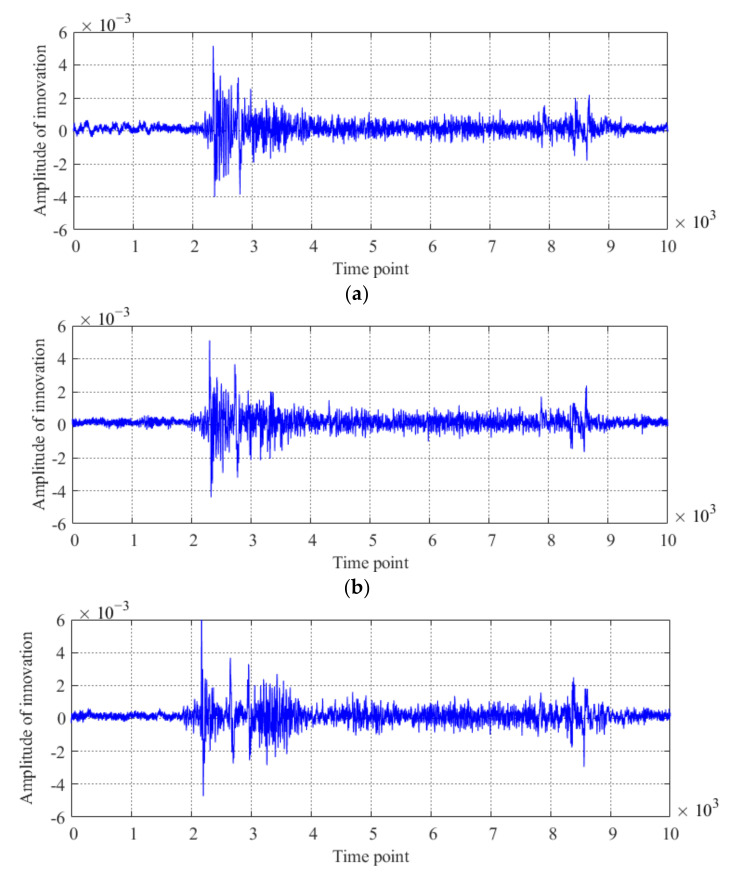

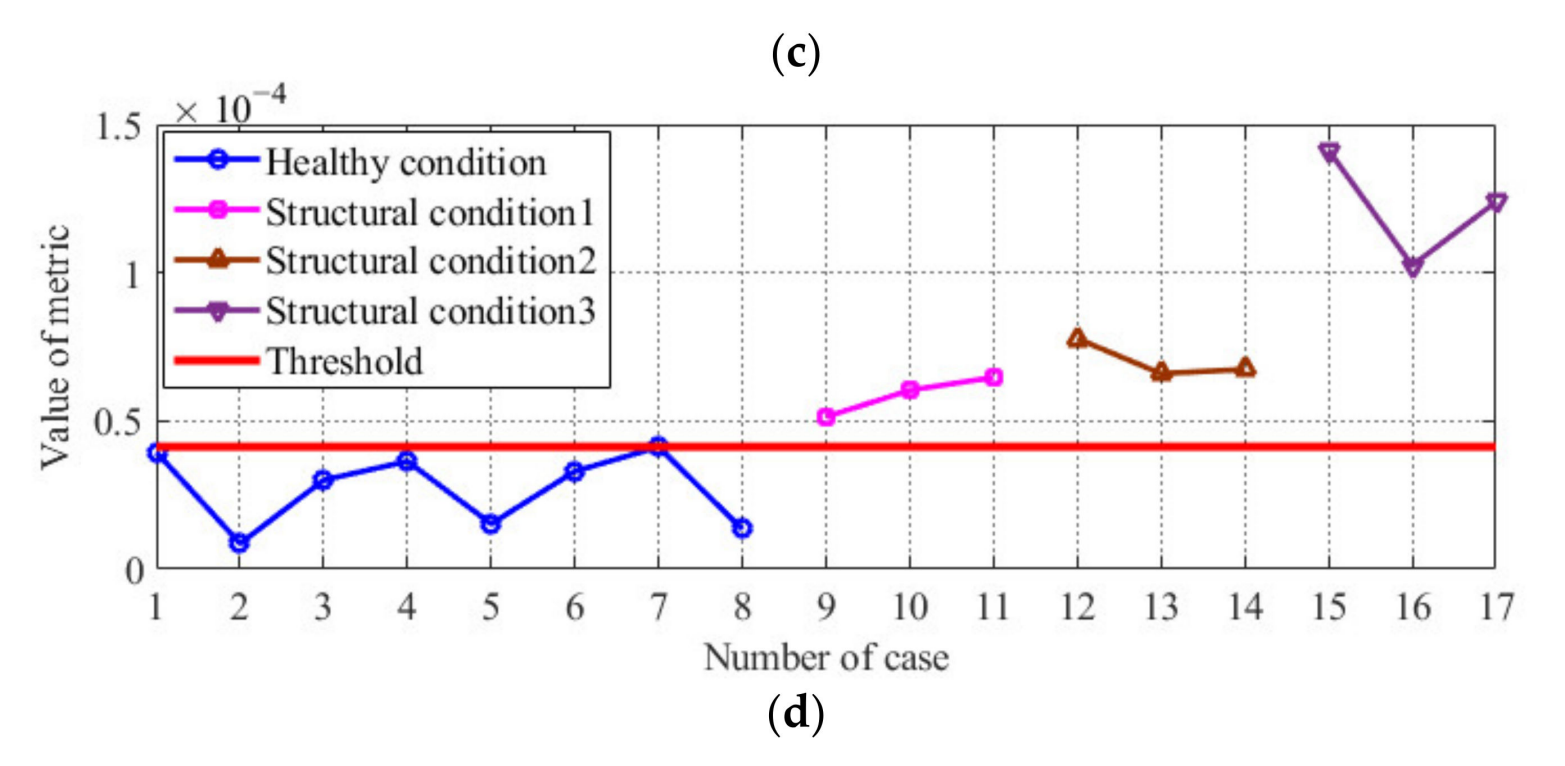
Results of condition diagnosis by using the proposed method: innovation of the #3 sensor for the model bridge under (**a**) structural condition 1; (**b**) structural condition 2; and (**c**) structural condition 3 and (**d**) condition diagnosis results for the model bridge.

Utilizing the generated Kalman filter, the condition diagnosis feature and condition diagnosis index are calculated with the procedure described in Figure 1. The threshold for judging the abnormal condition of the bridge is determined by using the acceleration data obtained from Case 2 through Case 10, as shown in Figure 6. For the different damage conditions of the model bridge, the innovations obtained by using the acceleration data of sensor #3 are shown in Figure 6a–c. The results of condition diagnosis are shown in Figure 6d.

As shown in Figure 6d, for three different damage conditions of the model bridge, there are three cases for each damage condition, and the values of the condition diagnosis index for the three damage conditions of the model bridge are all larger than the threshold. It is obvious that structural condition 3 is the most novel among the three abnormal conditions of the model bridge, which is consistent with the real situation because the damage extent of the model bridge under structural condition 3 is more severe than that under the other two structural conditions. The abovementioned results show that the proposed method is effective for diagnosing the abnormal condition of a bridge.

### 3.3. Comparison of the Performance between the Proposed Method and the Method Based on Modal Parameters

**Figure 7:**
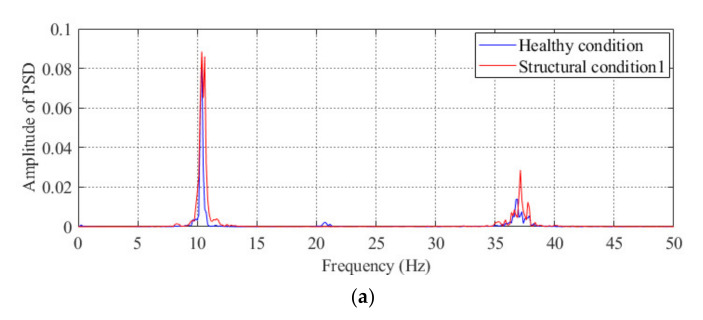

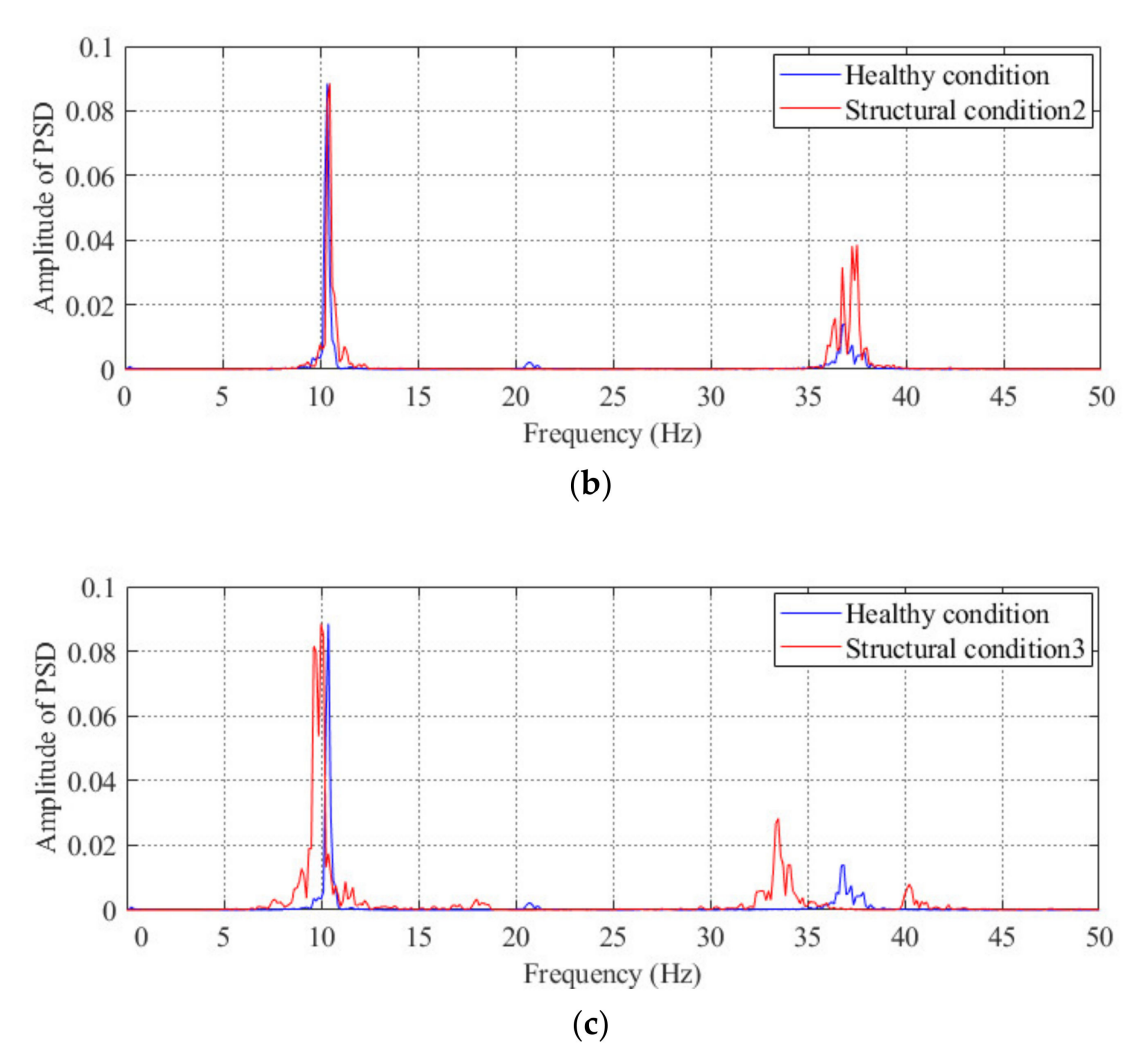
Auto-PSD obtained by using the acceleration response of sensor #3 under different structural conditions of the model bridge: comparisons between the healthy condition and (**a**) structural condition 1; (**b**) structural condition 2, and (**c**) structural condition 3.

After demonstrating the effectiveness of the proposed method, the performance of the proposed method is compared with a popular method based on modal parameters in this section. For three different damage conditions of the model bridge, the power spectral density (PSD) of the acceleration obtained from sensor #3 is shown in Figure 7. From the finite element analysis, we know that the first natural frequency of this model bridge is approximately 10 Hz; thus, the frequency of the first peak shown in the PSD figure is believed to be the measured natural frequency of the model bridge. As shown in Figure 7c, the abnormal condition of the model bridge under structural condition 3 can be effectively diagnosed from to the obvious difference in the first peak between the healthy condition and structural condition 3. However, it is difficult to diagnose the abnormal condition of the model bridge under the other two damage conditions by using the method based on modal parameters. As shown in Figure 6, the two abovementioned conditions can be diagnosed by using the proposed method. Therefore, compared with the traditional method based on modal parameters, the proposed method is more sensitive to structural conditions with minor damage.

### 3.4. Influence of Different Moving Loads on the Results of Condition Diagnosis of the Model Bridge

**Figure 8:**
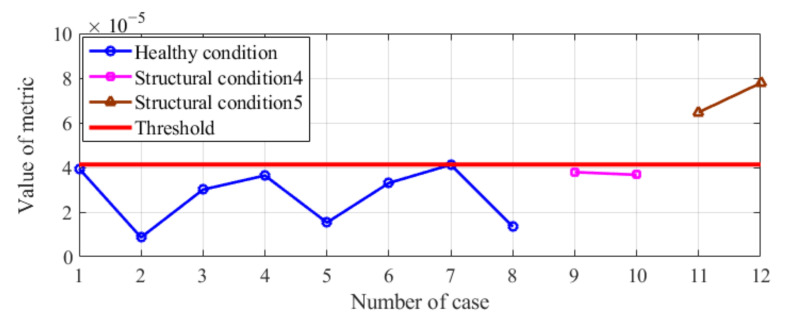
Results of condition diagnosis of the model bridge excited by different moving loads.

In this section, we verified whether the proposed method was effective in dealing with a disturbance or noise in the excitation load. As shown in Figure 8, when the difference in vehicle weight between the healthy condition and the condition to be diagnosed is less than 10% (e.g., structural condition 4), there is no misjudgment of the diagnosed results. However, when this difference is greater than 10% (e.g., structural condition 5, wherein the difference is approximately 20%), the obtained results are clearly incorrect. Therefore, if the disturbance or noise in the excitation load between the healthy condition and the abnormal condition is excessively large, the proposed method may fail to diagnose the condition of the bridge.

To discuss the reason for the above phenomenon, we need to analyze the effect of measured noise on the innovation obtained by the Kalman filter. According to Equations (3) and (4), the covariance of ${\left\{w_{k},v_{k}\right\}}^{T}$ is defined by the following equation:

(22) $$E\left\{\begin{array}{c} w_{k}\\ v_{k} \end{array}\right\}\left\{w_{k}{}^{T},v_{k}{}^{T}\right\}=\left\{\begin{array}{cc} Q_{k} & S_{k}\\ S_{k}{}^{T} & R_{k} \end{array}\right\},$$

where Q and R are the covariance matrixes of the disturbance of the excitation load and the measured noise, respectively. For actual bridges, $\rho_{k}$ and $\eta_{k}$ described in Equations (1) and (2) are always uncorrelated. Hence, the following relationships are obtained:

(23) $$Q=GQ_{b}G^{T},$$

(24) $$S=GQ_{b}D^{T},$$

(25) $$R=DQ_{b}D^{T}+R_{b},$$

(26) $$Q_{b}=E(\rho_{k}\rho_{k}{}^{T}),$$

(27) $$R_{b}=E(\eta_{k}\eta_{k}{}^{T}).$$

As discussed in [23], the correlation of innovations obtained by the Kalman filter depends on the structural damage of the bridge and the changes in $Q_{b}$ and $R_{b}$. Therefore, the effect caused by $Q_{b}$ and $R_{b}$ should be controlled to a lower level to ensure that the innovation is sufficiently sensitive to the variation in structural condition. On this basis, the limitation of the proposed method is keeping the load consistent in each load test, which means keeping the weight, speed and driving route of the loading vehicle as constant as possible. According to our experience, the weight difference of the moving vehicle between two loading cases should be less than 10%.

## 4. Example of an Actual Bridge

### 4.1. Description of the Selected Bridge and Corresponding Load Test

**Figure 9:**
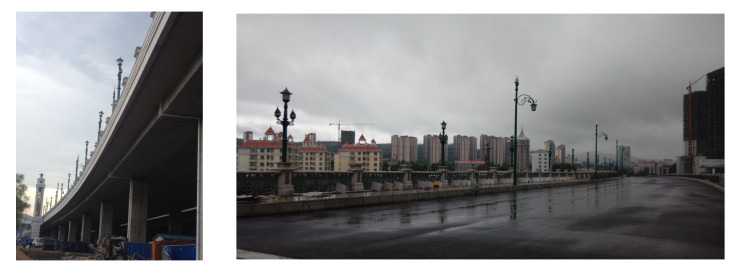
Photograph of the Xinwohong Bridge.

**Figure 10:**
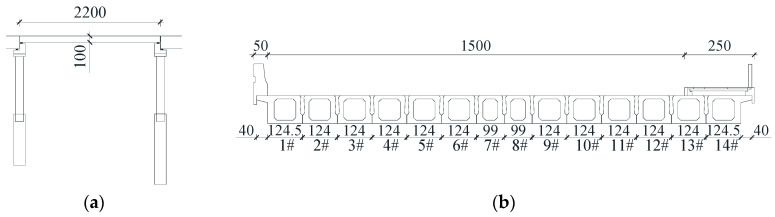
Detailed geometrical parameters of the actual beam bridge (units: cm): (**a**) vertical view and (**b**) cross-sectional view.

The Xinwohong Bridge, which is located in Shuangyashan, China, was taken as an example to evaluate the effectiveness of the proposed method. This bridge consists of two continuous beam spans and one simply supported beam span, as shown in Figure 9. The simply supported beam span was used in the load test following the proposed method. The detailed dimensions of this bridge are shown in Figure 10.

**Figure 11:**
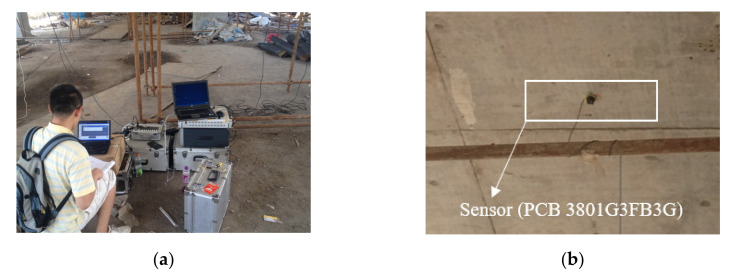
Photographs of the (**a**) data acquisition device and (**b**) accelerometers used for the load test of the actual beam bridge.

**Figure 12:**
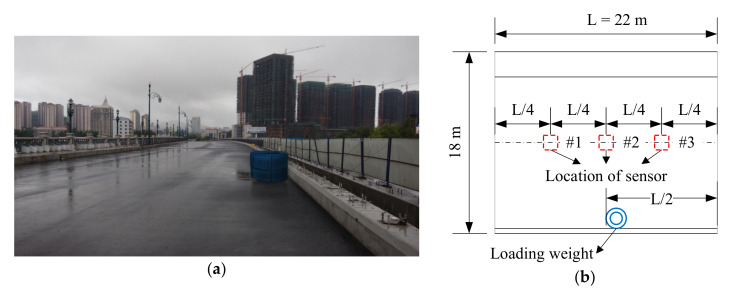

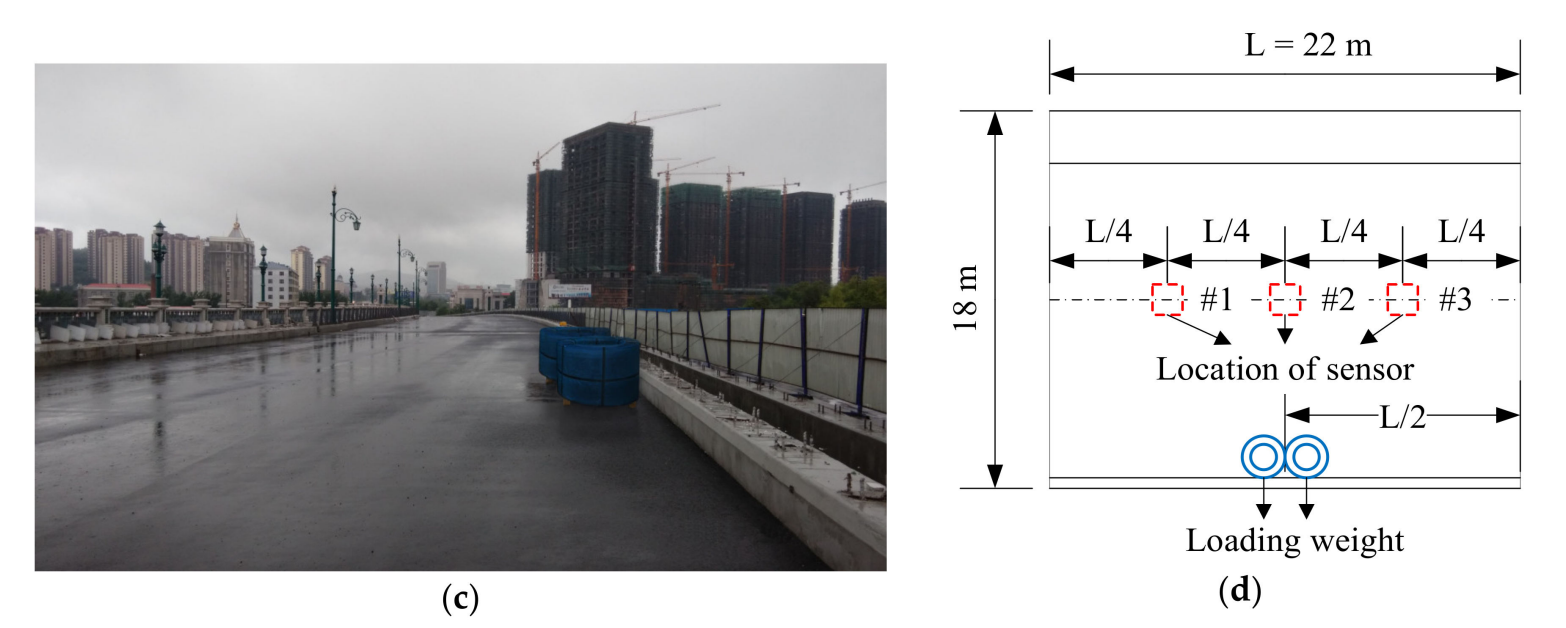
Photographs and placement of the steel weights and accelerometers for the actual beam bridge: (**a**) photograph of the bridge loaded with one steel weight; (**b**) placement of one steel weight and accelerometers; (**c**) photograph of the bridge loaded with two steel weights, and (**d**) placement of two steel weight and accelerometers.

The superstructure of this bridge is composed of 14 hollow slab prestressed concrete beams (Young’s modulus = 3.45 × 10^10^ Pa), and the cross-section of the superstructure is shown in Figure 10b. During the load test, three accelerometers (PCB Group, Inc., Depew, NY, USA) were installed on the bottom of the beam. Photographs of these sensors and schematics of the sensor placement are shown in Figure 11b and Figure 12b,d. The same data acquisition device used in the model test was applied to obtain the acceleration response of the superstructure of this bridge, and an on-site photograph is shown in Figure 11a. The load test was implemented when this bridge was just completed and ready to be opened to the public. The load test under the original condition of this bridge was repeated six times. To simulate the change in structural condition from the original condition, two additional steel weights, which were approximately 3 tons each, were placed on the surface of the bridge deck to create two new conditions (structural condition 1 and structural condition 2), as shown in Figure 12.

### 4.2. Results of Condition Diagnosis for the Actual Beam Bridge

**Figure 13:**
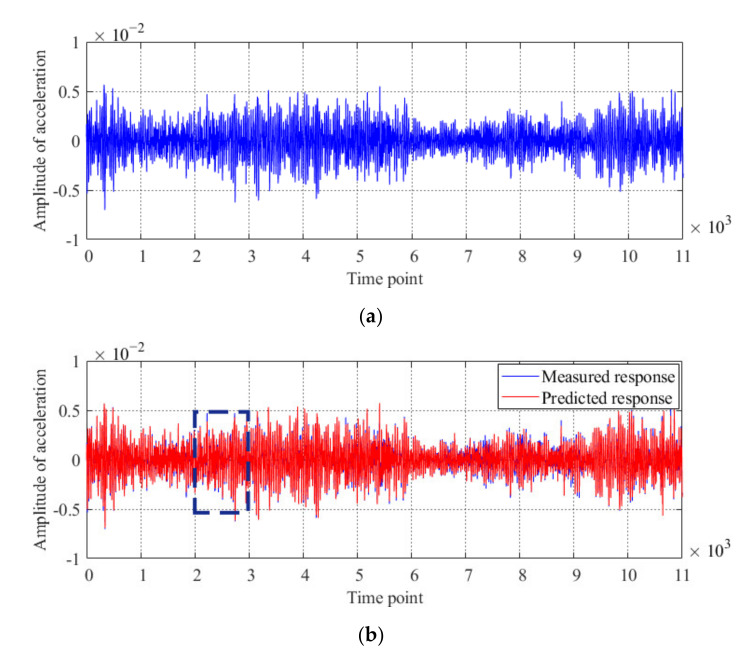

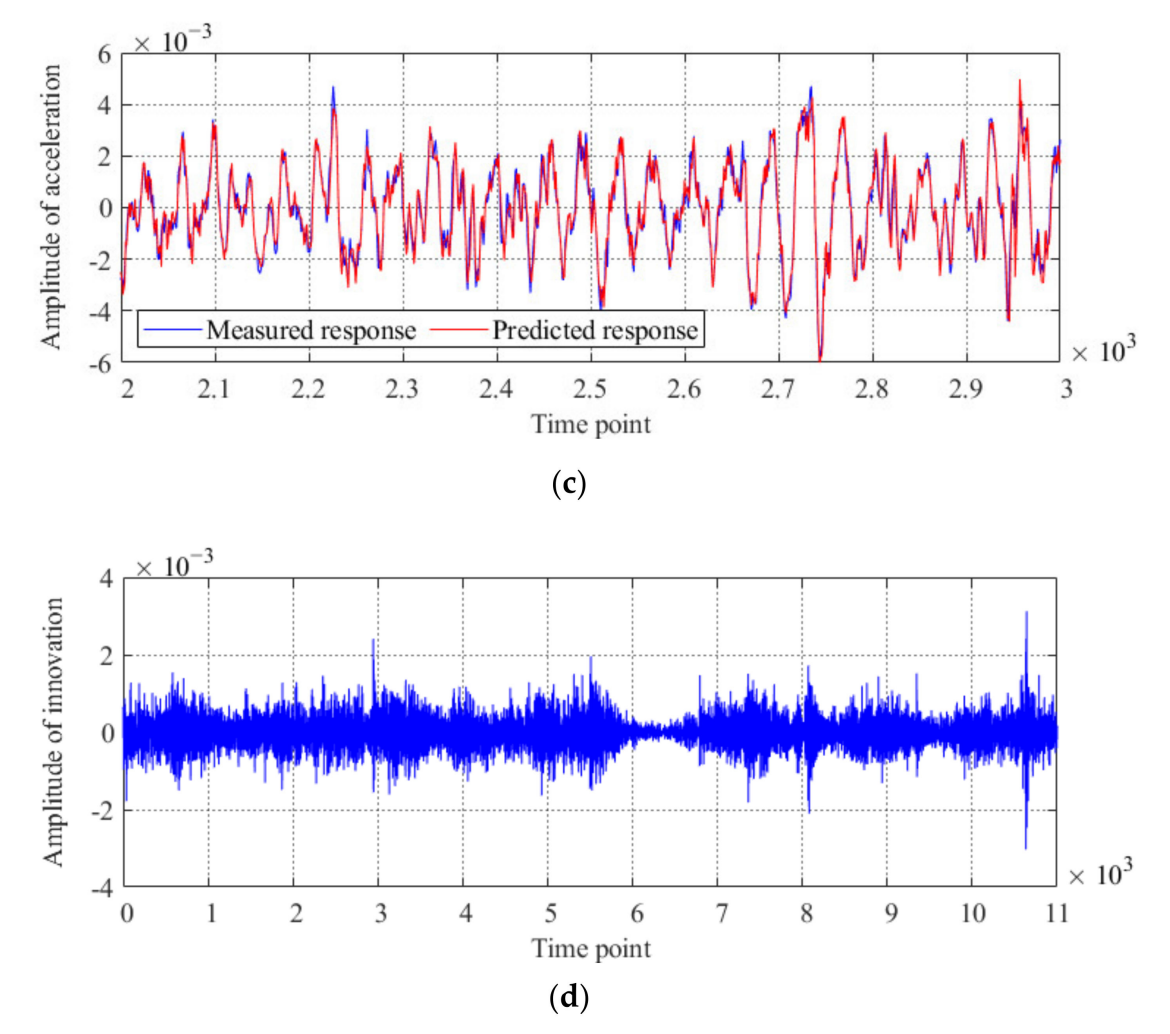
Results of the Kalman filter obtained by using the acceleration response of the actual bridge obtained under the first case: (**a**) measured acceleration response of sensor #2; (**b**) comparison of the measured and predicted acceleration responses of sensor #2; (**c**) detailed parts of the innovation from time points 2000 to 3000, and (**d**) innovation obtained by using the acceleration response of sensor #2.

Using a similar method as that used for the model bridge, all the acceleration responses obtained from the first case are implemented to generate the Kalman filter of the original condition of the actual bridge. The sampling frequency is set to 400 Hz, and the acceleration time history of the first 11,000 time point measured from sensor #2 is shown in Figure 13a. With the generated Kalman filter, the predicted acceleration is obtained, and a comparison between the predicted and measured accelerations is shown in Figure 13b–c. The innovation between the filter predictions and measured acceleration responses of sensor #2 is shown in Figure 13d.

**Figure 14:**
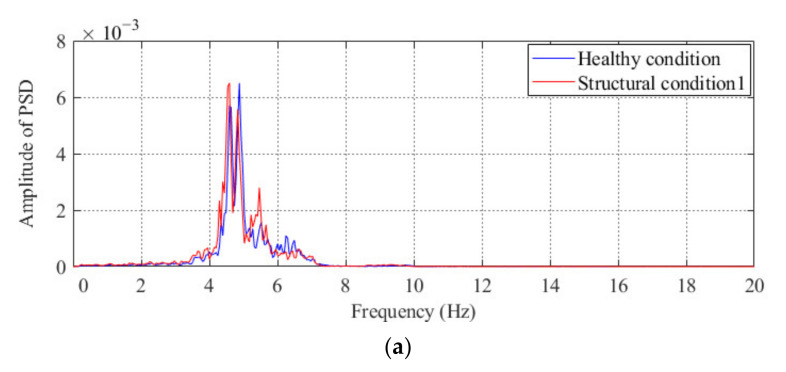

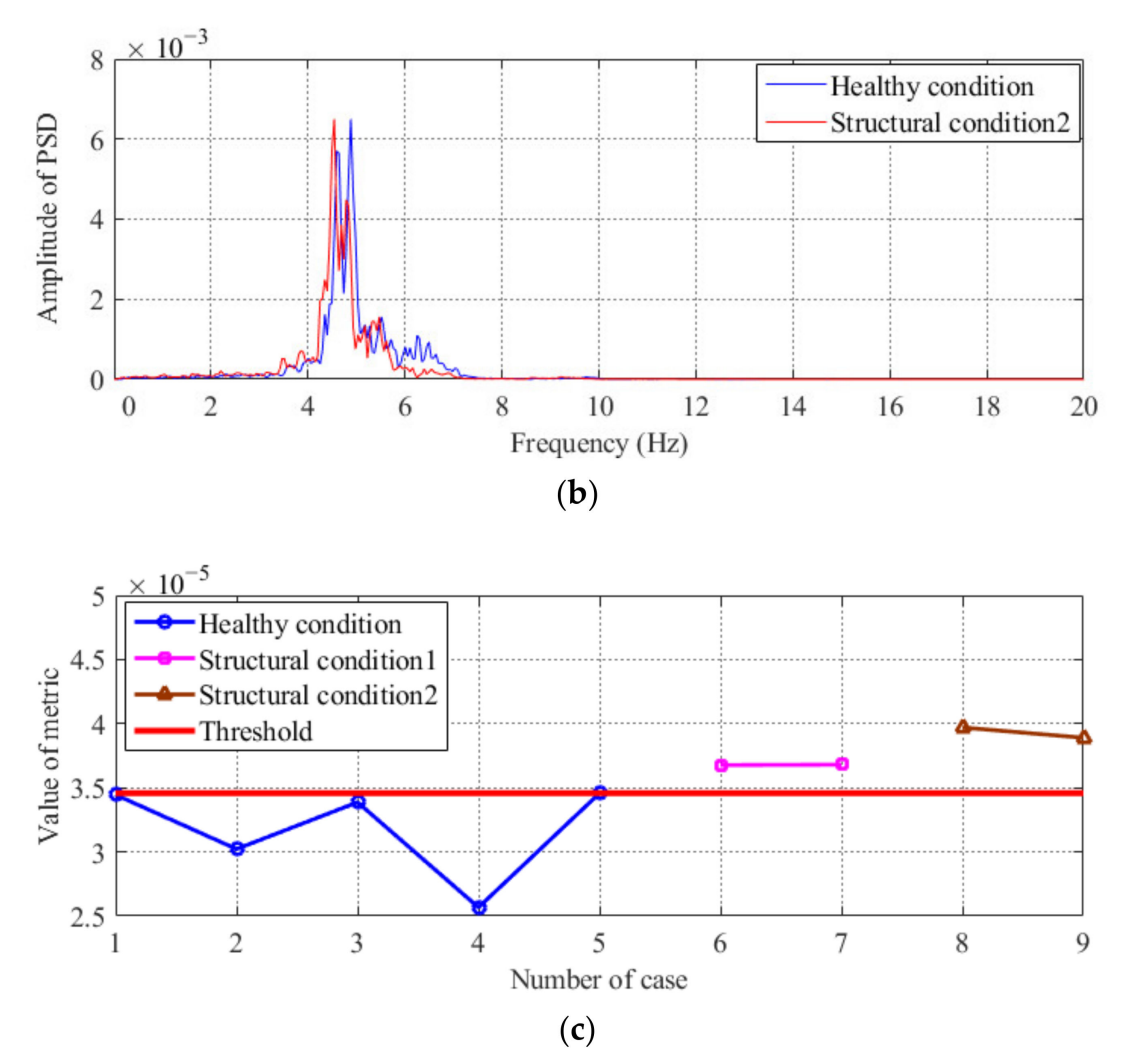
Results of condition diagnosis for the actual bridge by using the proposed method: auto-PSD obtained by using the acceleration response of the #2 sensor under (**a**) structural condition 1 and (**b**) structural condition 2 and (**c**) condition diagnosis results for the actual beam bridge.

Using finite element analysis, the first natural frequency of this bridge is 4.30 Hz, so the frequency of the first peak shown in the PSD plot is believed to be the measured natural frequency of the actual bridge, as shown in Figure 14a,b. Of course, it is difficult to diagnose the occurrence of abnormal conditions under structural condition 1 or structural condition 2 by directly using the method based on modal parameters. Compared with the modal parameters-based method, the proposed method can diagnose the change in the structural condition of this bridge, as shown in Figure 14c.

## 5. Conclusions

In this study, a Kalman filter-based method is proposed to diagnose the condition of medium- and small-span beam bridges during a brief interruption in traffic flow. The following conclusions are drawn from this study:

(1) The proposed Kalman filter-based method is suitable to diagnose the structural condition of bridges without any long-duration interruption of the traffic flow, and this method is convenient for practical application since its does not need to establish the FEM of the bridge.
(2) There is good agreement between the predicted and measured acceleration responses, which shows that the performance of Kalman filter is sufficient to predict the dynamic response of actual bridges excited by a moving load.
(3) A condition diagnosis index based on the energy ratio between the innovation obtained by the Kalman filter and the measured acceleration is proposed. The results of condition diagnosis using data from experiments and field tests show that this index is sensitive to changes in the structural condition of the bridge superstructure, thereby illustrating that the proposed index is suitable for evaluating the condition of actual medium- and small-span beam bridges.
(4) The results obtained from a model bridge and an actual bridge demonstrate that, in comparison with the traditional method based on modal parameters, the proposed method is more sensitive to the changes in the structural condition of bridges.
(5) The limitation of the proposed method in application is keeping the load consistent for each load test, and the difference in the weight difference of the moving vehicle between each test should be less than 10%.

## Figures and Tables

**Table 1:** Description of all cases for the experimental example.

Case Number	Description of Case
1–9	Healthy condition: bridge without any damage excited by a 120 kg moving load
10–12	Structural condition 1: damaged bridge (removing the #17 transverse connection) excited by a 120 kg moving load
13–15	Structural condition 2: damaged bridge (removing the #17 and #6 transverse connections) excited by a 120 kg moving load
16–18	Structural condition 3: damaged bridge (removing the #17, #6, #5, and #16 transverse connections) excited by a 120 kg moving load
19–20	Structural condition 4: bridge without any damage excited by a 130 kg moving load
21–22	Structural condition 5: bridge without any damage excited by a 140 kg moving load
